# Implementation of a Resident-Designed Procedure Cart in a Busy Emergency Department

**DOI:** 10.7759/cureus.42211

**Published:** 2023-07-20

**Authors:** Kimberly Johnson, Caroline Smith, Lee G Barker, Stephanie Iken, Jean Laubinger, Connor Fraser, Michael Falgiani, Leoh Leon, Samyr Elbadri, Joshua Walker, James L Wilson, Frank Fraunfelter, Latha Ganti

**Affiliations:** 1 Emergency Medicine, Hospital Corporation of America (HCA) Florida Ocala Hospital, Ocala, USA; 2 Emergency Medicine Residency, Hospital Corporation of America (HCA) Florida Ocala Hospital, Ocala, USA; 3 Emergency Medicine, Hospital Corporation of America (HCA) Ocala Hospital Florida, Ocala, USA; 4 Emergency Medicine, University of Central Florida College of Medicine, Orlando, USA; 5 Emergency Medicine, Envision Physician Services, Plantation, USA

**Keywords:** emergency medicine resuscitation, surgical supplies, quality improvement tool, emergency department, resuscitation, procedure cart

## Abstract

The authors present a description of the procedure cart they designed for their Emergency Department. This project was in response to the inefficiencies in having to gather supplies from various locations to get set up. A complete description including each of the drawer contents is provided to allow others to easily replicate a tool that saved the authors much time and frustration in daily practice.

## Introduction

Emergency medicine is full of critical decisions and quick interventions in the stabilization of the undifferentiated, often critically ill patient. Emergency Department physicians are frequently pressed for time with performing the many diverse aspects of patient care. There are many instances where short periods of time can significantly alter patient outcomes. Physicians in the Emergency Department must perform multiple procedures daily to help stabilize patients, protect their airways, or administer life-saving medications. These procedures need to be completed quickly and efficiently to ensure appropriate lifesaving care.

A group of emergency medicine residents at the HCA Florida Ocala Hospital noted a significant barrier to patient care with regard to critical procedures. Due to the design of the constantly expanding Emergency Department (ED), there were multiple supply closets where materials for procedures had to be gathered. This team noted they would have to visit approximately two to four supply closets to gather the appropriate materials for commonly performed procedures such as central and arterial lines. Decentralized supply storage has been shown to significantly delay emergency procedures such as ventriculostomy for example [1]. After several months of struggling with this inefficient system, this team proposed the idea of a procedure cart to the Emergency Department staff and administration.

## Technical report

When designing the procedure cart, we surveyed resident physicians and nursing staff to demonstrate the utility a cart would provide. Out of 39 respondents, 77% reported taking longer than 5 min to gather supplies and personal protective equipment for critical procedures; 82% said they went to two separate supply locations; and 0% said that procedure supplies were available in patient rooms.

**Figure 1:**
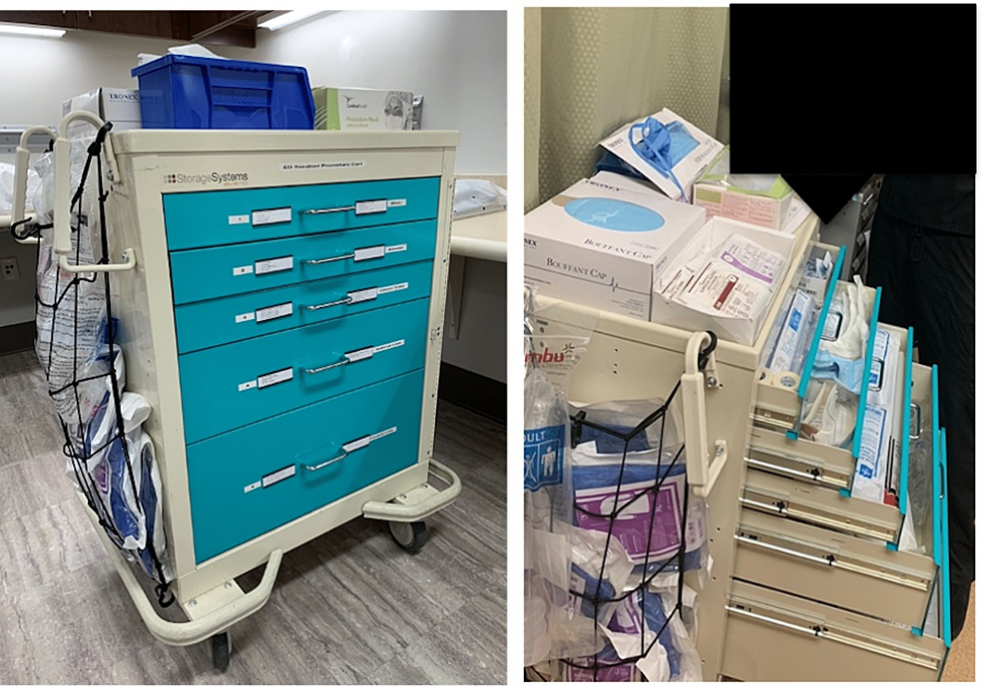
Procedure cart.

We wanted our procedure cart to follow a design and protocol similar to that of a code cart [2]. We chose a cart of similar size, although teal in color, to easily distinguish it from a code cart. In addition, the space on the top of the cart and the side of the cart, was utilized for bulkier items such as personal protective equipment (PPE) and bag-valve-mask (BVM). There are five central drawers, most organized by procedure type for easy accessibility (Figure 1).

Table 1 lists the contents of the drawers.

**Table 1:** Contents of the procedure cart. BVM, bag-valve-mask; CVC, central venous catheter; OG, orogastric; ET, endotracheal tube

Quantity		Item
Top and sides of the cart		
1	Surgical caps	
1	Surgical gloves (place in box)	
1	Face masks	
	Gowns (side of cart)	
1	BVM (side of cart)	
1	Purple top cleaning wipes	
Drawer 1		
10	Central line caps (place in box)	
8	Biopatches (place in box)	
8	US probe covers	
2	1 povidone-iodine and 1 chlorhexidine solution	
2	Silk tape	
2	Self-adherent wrap	
Fill space	Arterial arrows	
10	Transparent dressing (large and small)	
6	Gauze boats	
5	Surgical prepping solution	
5	Size 0 perma-hand silk straight needle suture	
5	Size 0 perma-hand silk curved needle suture	
Drawer 2		
2 2 1 1	8.0 ET tube 7.5 ET tube 7.0 ET tube 6.5 ET tube	
1	DL Bag - Handle - +C Batteries (x2) - Mac blades 3 & 4 - Miller blades 2, 3, & 4	
1	+ CO2 detector	
1	Bougie	
	OPA/NPA box screw, NPA, Bite block, OPA (8,9 & 10)	
1	PEEP valve	
1	Ostomy	
1	Blades 3 and 4	
1	DL stylet	
1	Rigid stylet	
Drawer 3		
2	Large blue sterile towels	
1	Disposable chest tube kit	
6	Pre-cut drain sponges	
6	Large occlusive dressing	
1	25 cm 10 Fr pigtail catheter	
1	25 cm 8.5 Fr pigtail catheter	
2	32 Fr chest tube	
2	28 Fr chest tube	
2	4 Fr chest tube	
2	20 Fr chest tube	
4	14 Ga needle for aspiration	
Drawer 4		
4	A-Line tubing	
2	Femoral arterial line kit	
4	Wrist assist	
4	500 mL pressure bag	
4	500 mL NS	
4	A-Line cable for monitor	
2	Large blue sterile towels (if they cannot fit in drawer 3)	
4	Surgical prepping solution	
Drawer 5		
2	16 cm triple lumen 7 Fr CVC kit	
2	20 cm triple lumen 7 Fr CVC kit	
1	IO kit with drill and needles	
1	9 fr double lumen cordis CVC kit	
-	Miscellaneous supplies (suction tubing, OG tubing, etc.)	

The first drawer is miscellaneous, although important things include the frequently used small blue caps for central lines and ultrasound probe covers. The second drawer contains all the materials needed for intubation and airway management (with the exception of the ventilator). The third drawer contains needle decompression and chest tube supplies. The fourth drawer contains the necessary supplies for placing arterial lines and the entire arterial line setup. The bottom drawer mainly consists of central line kits of multiple sizes as well as an intraosseous access kit. We also noted a need for easily accessible personal protective equipment. Therefore, the top of the cart and the side of the cart have gloves, sterile gloves, surgical caps, and gowns.

Similar to how the code carts in our Emergency Department are checked daily, we wanted a scheduled restocking system for our procedure cart. The procedure cart was determined to be organized and restocked according to the standard checklist once every 24 h. This has been determined to be done during the night shift, which has led to better compliance as one shift is now responsible for restocking. 

Since implementation, our procedure cart has successfully minimized barriers to performing critical patient procedures without delay. Upon arrival of a critical patient, staff can bring the procedure cart to the room, providing immediate access to supplies at the bedside. Since improving efficiency and patient care in the Emergency Department at our hospital, similar procedure carts have been designed and implemented in other Emergency Departments within our health system.

## Discussion

Timeliness is critical to improving outcomes in the Emergency Department. For example, every minute delay in cardiac resuscitation decreases the successful return of spontaneous circulation (ROSC) by 5% [3]. Easily accessible procedure carts improve the speed of central lines, chest tubes, and other standard critical resuscitative measures in the Emergency Department. This applies both to large Emergency Departments with multiple stock rooms in various stages of resupply and to free-standing Emergency Departments with more limited staffing.

It is essential that the procedure carts remain well-stocked. To that end, new staff should be formally oriented to the purpose, location, and method of restocking the procedure cart. For our Emergency Department, the responsibility falls once per day to the night shift and to providers who notice the absence of resources following multiple resuscitations during a single shift. We recommend that problems with the resupply of the carts by designated staff be communicated to physicians and nursing leadership.

Furthermore, the placement of procedure carts within the Emergency Department environment is critical to their successful utilization. Most Emergency Departments group high-acuity patients in adjacent rooms or within the same hall. We recommend keeping the procedure in a fixed location near the high-acuity rooms. In the event of a resuscitation outside of the high acuity area, the cart should be brought back promptly to the hall or alcove following the resuscitation. 

Staff should remain oriented to the precise location of BVMs, endotracheal tubes, pigtail catheters, etc. within the supply closets if the procedure cart does not have the proper equipment. It is also not uncommon for an Emergency Department to have multiple specialized procedure carts including obstetrics, pediatrics, urology, and orthopedics. We recommend clear, intuitive labeling of carts with color-coded, large font be employed to avoid incorrect identification.

Procedure carts can be useful outside of the Emergency Department as well. A report of such a cart in an internal medicine academic practice reported increased frequency of use, satisfaction, and perceived improvement in efficiency and patient safety [4]. Increased efficiency in the form of decreased consult-to-patient treatment time was also reported following the implementation of a plastic surgery specialty cart [5].

## Conclusions

The design and employment of a crash procedure cart in our Emergency Department has improved the timely resuscitation of unstable patients. The degree to which it has quantitatively improved the speed of resuscitation, patient outcomes, and time to disposition remains unmeasured. Further investigation into these quality metrics will be the next step in optimizing the materials, location, and number of procedure carts in the Emergency Department. 
